# Celecoxib Enhances the Efficacy of Low-Dose Antibiotic Treatment against Polymicrobial Sepsis in Mice and Clinical Isolates of ESKAPE Pathogens

**DOI:** 10.3389/fmicb.2017.00805

**Published:** 2017-05-08

**Authors:** Madhavi Annamanedi, Gajapati Y. N. Varma, K. Anuradha, Arunasree M. Kalle

**Affiliations:** ^1^Department of Animal Biology, School of Life Sciences, University of HyderabadHyderabad, India; ^2^Pathology and Lab Medicine, Asian Institute of GastroenterologyHyderabad, India

**Keywords:** antibiotic drug resistance, celecoxib, polymicrobial sepsis, ESKAPE pathogens, imipenem, ampicillin

## Abstract

Treatment of multidrug resistant bacterial infections has been a great challenge globally. Previous studies including our study have highlighted the use of celecoxib, a non-steroidal anti-inflammatory drug in combination with antibiotic has decreased the minimal inhibitory concentration to limit *Staphylococcus aureus* infection. However, the efficacy of this combinatorial treatment against various pathogenic bacteria is not determined. Therefore, we have evaluated the potential use of celecoxib in combination with low doses of antibiotic in limiting Gram-positive and Gram-negative bacteria *in vivo* in murine polymicrobial sepsis developed by cecum ligation and puncture (CLP) method and against clinically isolated human ESKAPE pathogens (*Enterococcus faecium, Staphylococcus aureus, Klebsiella pneumoniae, Acinetobacter baumannii, Pseudomonas aeruginosa*, and *Enterobacter* species). The *in vivo* results clearly demonstrated a significant reduction in the bacterial load in different organs and in the inflammatory markers such as COX-2 and NF-κB *via* activation of SIRT1 in mice treated with imipenem, a choice of antibiotic for polymicrobial sepsis treatment. Combinatorial treatment of ampicillin and celecoxib was effective on clinical isolates of ESKAPE pathogens, 45% of tested clinical isolates showed more than 50% reduction in the colony forming units when compared to ampicillin alone. In conclusion, this non-traditional treatment strategy might be effective in clinic to reduce the dose of antibiotic to treat drug-resistant bacterial infections.

## Introduction

Multidrug resistance is an adaptation of bacteria for its survival (Sorlozano et al., 2014). Bacteria are evolving rapidly challenging the current antibiotic treatment strategies. The cost involved in the treatment of drug-resistant bacterial infections such as methicillin-resistant *Staphylococcus aureus* (MRSA) is a burden to both patient and the country (Filice et al., 2010). Furthermore, the cost and time involved in the development of new drug for the treatment of such “superbugs” is very huge which is soon followed by the development of resistance to the new antibiotic. In this context, finding a non-traditional treatment strategy consisting of combination of an antibiotic targeting bacteria and a non-antibiotic agent that does not have any target in bacteria but helps in improving host response or potentiating antibiotic activity even at low doses of antibiotic is preferred (Wang et al., 2015).

Celecoxib is a Cyclooxygenase-2 (COX-2) specific inhibitor approved by FDA for the treatment of rheumatoid arthritis. COX-2 is induced by inflammatory stimuli and is responsible for the prostaglandins (PGE2) production at the site of inflammation (Ricciotti and FitzGerald, 2011). During infection, functions like phagocytosis and bacterial killing are inhibited by excessive production of PGE2. Therefore, administration of COX-2-specific inhibitor such as celecoxib might reduce the production of PGE2 and inflammation and stimulates phagocytosis (Aronoff et al., 2005). Previously, we have demonstrated that celecoxib increased the sensitivity of laboratory strains of both Gram-positive and Gram-negative bacteria to antibiotics at half their minimal inhibitory concentrations (MIC) (Kalle and Rizvi, 2011). Next, we have also evaluated the efficacy of this combinatorial treatment on intracellular RAW 264.7 macrophage-phagocytosed *S aureus* (Annamanedi and Kalle, 2014). The results have clearly demonstrated a significant reduction in the bacterial load in combinatorial treatment along with significant decrease in inflammatory markers. A recent study by Thangamani et al. (2015) have also clearly demonstrated the efficacy of celecoxib against *S aureus*. However, the efficacy of such treatment strategy on various pathogens causing clinical and economic burden needs to be evaluated.

The present study was done to evaluate the efficacy of this non-traditional treatment strategy of celecoxib and antibiotic combination against various pathogenic bacteria using murine model of polymicrobial sepsis by cecal ligation and puncture (CLP) method. The cecum has been highly colonized with microbes, generally huge number of Gram-negative and Gram-positive bacteria, puncture of the cecum releases fecal material into the peritoneal cavity to generate an excessive immune response brought by polymicrobes present in the cecum (Toscano et al., 2011). Imipenem was choice of antibiotic to treat septic mice in combination with celecoxib as imipenem is clinically effective in treating various types of Gram-negative and/or Gram-positive bacterial infections and is often recommended for the treatment of polymicrobial intra-abdominal infections (Rodloff et al., 2006). Imipenem belongs to carbapenems class of antibiotics and is a β-lactam antibiotic.

ESKAPE, an acronym for *Enterococcus faecium, Staphylococcus aureus, Klebsiella pneumoniae, Acinetobacter baumannii, Pseudomonas aeruginosa, and Enterobacter* species, pathogens are the global emerging cause of nosocomial infections and are multidrug resistant (Santajit and Indrawattana, 2016). The currently available antibiotics are not effective against some highly resistant ESKAPE pathogens such as *Acinetobacter* species, MDR- *P. aeruginosa*, *Klebsiella* species, and *E. coli* which led to the use of old antibiotics like colistin that are associated with severe toxicity (Boucher et al., 2009). The present study, thus, has also evaluated the efficacy of ampicillin and celecoxib on the growth of clinical isolates of drug resistant ESKAPE pathogens. Ampicillin was the choice of antibiotic for the clinical isolates due to its wider availability and cost effectiveness.

The present study, therefore, evaluated the efficacy of the promising non-traditional antibacterial therapy using the combination of antibiotic and celecoxib in mouse model of sepsis and on clinical isolates.

## Materials and Methods

### *In Vivo* Experiments

#### Reagents and Antibodies

Celecoxib was obtained from Aurobindo Pharma Ltd., India. Imipenem and ampicillin was purchased from Sigma-Aldrich, USA. All the primary and secondary antibodies used were purchased from Abcam, UK.

#### Mice and Infection

Animal experiment was conducted with approval from Institutional Animal Ethics Committee (UH/IAEC/AMK/2011-II/5). Female BALB/c mice weighing 19–20 g were procured from National Institute of Nutrition, India. Mice were divided into five groups (*n* = 5 in each group), sham group (taking cecum out and replaced back but neither ligated nor punctured), CLP group (control mice group), imipenem treated, celecoxib treated and both imipenem and celecoxib treated group. Following the protocol described by Toscano et al. (2011), sepsis by CLP was developed in mice. Briefly, the mice were anesthetized with ketamine (75 mg/kg) and xylazine (15 mg/kg), and a 2-cm midline incision was made through the linea alba. The cecum was located and ligated with sterile 3-0 silk, and perforated with double puncture using a 20-gauge needle. To make sure that the cecum is punctured and conditions for septicemia are created, a small amount of cecum contents are extruded out. Then the cecum was replaced in its original position within the abdomen, and incision was closed immediately.

#### Treatment

Soon after surgery, each mouse has received a subcutaneous injection of 1 ml of warm (37°C) normal saline with tramadol hydrochloride (analgesic) (20 mg/kg body weight). Test compound group mice [imipenem (5 mg/kg) or/and celecoxib (5 mg/kg)] received two subcutaneous injections of test compounds after 4 and 10 h of surgery. All the mice were kept at normal conditions with an extra vigilance. The mice were sacrificed after 10 h of last drug treatment. Organs such as liver, kidney, heart, lung, and spleen were collected by following standard procedures and stored at -80°C for further studies.

#### Gram Staining

A thin layer of liver or spleen tissue homogenate was spread onto a microscopic slide using 50 μl of sample and allowed to air dry. Then the slides were heat fixed for approximately 10 s and stained using Gram stain kit (Fisher Scientific, USA) as described earlier (Steinhauser et al., 1999). The staining was performed using homogenates of all mice in sham group and representative figures are given.

#### Bacterial Load Determination

The whole organ (spleen and liver) was placed in 900 μL of sterile PBS and then mechanically disrupted by macerating the organ with a sterile Pasteur pipette and then vortexing vigorously. Aliquots (100 μL) of supernatant from disrupted organs were plated on LB agar plates (Kokai-Kun et al., 2007) and incubated at 37°C for 18 h. The colonies were counted and a graph was plotted with CFU/gram tissue on *Y*-axis. Bacterial load was determined in spleen and liver of all five mice in a group and the graph represents the means ± SD of five mice.

#### Western Blot Analysis

Immunoblot analysis was carried out according to the standard procedure (Liu Z.Q. et al., 2014). The membranes were probed with 1:1000 dilution of anti-COX2 (Abcam, UK), anti-SIRT1 (Abcam, UK) and anti-β actin (Abcam, UK) antibodies. β-Actin served as loading control.

#### Analysis of Tissue mRNA Levels by Real-Time RT-PCR

Total RNA was extracted with TRIZOL (Sigma-Aldrich, USA) and 1 μg of RNA was reverse-transcribed with reverse transcription kit, (Invitrogen) as specified by the manufacturer. Real-time RT-PCR was performed on Applied Biosystems StepOnePlus^TM^ Instrument using KAPA SYBR^®^ FAST qPCR master mix and gene-specific primers (**Table 1**).

**Table 1:** Primers used for the quantitative real time PCR analysis of cytokines.

Gene	Forward primer	Reverse primer	Source
TNF-α	TTCTGTCTACTGAACTTCGGGGTGATCGGTCC	GTATGAGATAGCAAATCGGCTGACGGTGTGGG	Wang et al., 2013
IL-β	ATGGCAACTGTTCCTGAACTCAACT	CAGGACAGGTATAGATTCTTTCCTTT	Zeng et al., 2015
IL-6	AGGATACCACTCCCAACAGACCT	CAAGTGCATCATCGTTGTTCATAC	Zeng et al., 2015
IL-10	ATTTGAATTCCCTGGGTGAGAAG	CACAGGGGAGAAATCGATGACA	Yasuma et al., 2016


#### Measurement of p65 Levels of Nuclear Factor-κB (NF-κB)

NF-κB-p65 a subunit of NF-κB transcription factor complex which controls cytokine gene expression. Calculation of the nuclear p65 relative to cytoplasmic p65 concentration ratio estimates NF-κB activation. An ELISA based NF-κB p65 measuring kit (Cell Signaling Technologies, USA) was used as per manufacturer’s protocol to estimate NF-κB activation.

#### Peroxidase and Catalase Assay

Catalase and peroxidase activity in the whole cell lysates of spleen and liver was determined as previously described (Lefebre and Valvano, 2001).

### Clinical Isolates

#### Bacterial Strains Collection

Total of 507 bacterial isolates was collected from the Asian Institute of Gastroenterology, Hyderabad, India during the period of 2012 to 2013. Strain identification and antibiotic profiling was determined by analytical profile index (API) strips using VITEK 2 automated system (bioMérieux). Glycerol stocks of the isolates were prepared and stored at -80°C until use. Randomly, 32 isolates (*n* = 32) belonging to each group of ESKAPE pathogens were selected for the study except for *Staphylococcus aureus* where *n* = 24.

#### Determination of Colony Forming Units of ESKAPE Pathogens

Colony forming units of each of ESKAPE pathogens isolated from patients samples were determined by agar dilution method in duplicates, according to the CLSI protocol (Clinical and Laboratory Standards Institute, 2012). Briefly, two single isolated bacterial colonies were grown overnight on Muller Hilton (MH) agar plate (HIMEDIA, India) and was resuspended in MH broth, adjusted to 0.5 McFarland and diluted to get 10^4^ CFU/mL. Bacterial suspension was spread on to the agar plates containing 2 μg/ml of ampicillin and/or 3.8 μg/mL celecoxib. Plates were kept at 37°C for 24 h. Colonies formed on plates from initial two single colonies were counted and mean ± SD CFU/mL was calculated.

### Statistical Analysis

For *in vivo* experiments results were expressed means ± SD of five mice and mean ± SD for *in vitro* clinical isolates from duplicate values (**Supplementary Data Sheet 1**). The statistical analyses were performed using two-way analysis of variance (GraphPad Prism) and statistically significant differences were established as *p* < 0.05, indicated by ^∗^.

## Results

### Combination of Celecoxib and Imipenem Reduced the Bacterial Survival Load in Liver and Spleen

**FIGURE 1:**
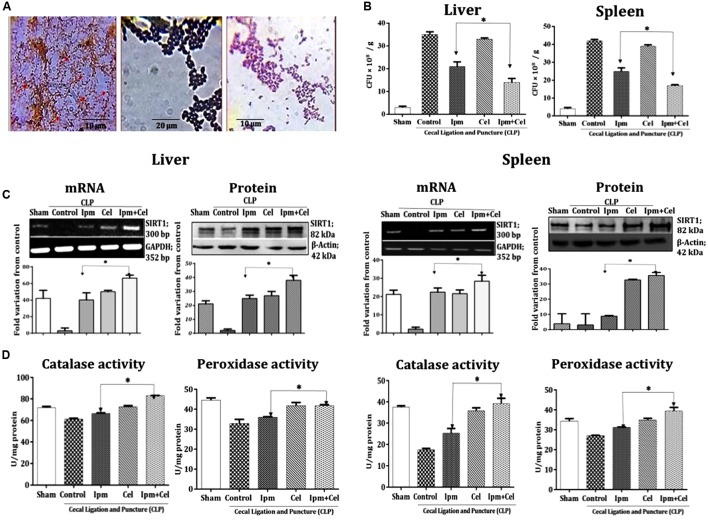
**Combination of celecoxib and imipenem reduced the bacterial survival load significantly in liver and spleen tissues of CLP mice than imipenem alone and the activation of SIRT1 by celecoxib increases antioxidant enzyme activity.** **(A)** Presence of bacteria in the different tissue homogenates of mice with CLP (right). Gram staining of isolated colonies formed on agar plates by spreading tissue homogenates showing Gram-positive bacteria (middle) and Gram-negative bacteria (left). **(B)** Effect of imipenem; IPM (5 mg/kg) celecoxib; CEL (5 mg/kg) and their combination on the survival of intracellular bacteria in the liver tissue and spleen tissue as measured by the CFU/g. **(C)** RT-PCR and Western blot analysis of SIRT1in Sham (Lane 1); CLP; (Lane 2); imipenem; IPM 5 mg/kg (Lane 3), celecoxib; CEL 5 mg/kg (Lane 4), and combination of celecoxib and imipenem (Lane 5) in the liver tissue (right), and spleen tissue (left), respectively. GAPDH and β-actin served as controls for RT-PCR and Western blot, respectively. The relative band intensities were measured by ImageJ software. **(D)** Activity assay for antioxidant enzymes, catalase, and peroxidise, was carried out in Liver tissue (right), and spleen tissue lysates (left). Bars indicate the mean ± SD (*n* = 5); CLP, cecal ligation and puncture. ^∗^*p* < 0.05.

To evaluate the potency of celecoxib and antibiotic combination therapy *in vivo*, we used mouse model of sepsis by CLP. First to confirm the presence of bacterial load in various organs of CLP mice, Gram staining was carried out using liver and spleen tissue homogenate and the results indicated the presence of both Gram-positive and Gram-negative bacteria and most of them were cocci (**Figure 1A**). Next, we tried to quantify the bacterial load in liver and spleen by counting CFU on agar plates. The results indicated that there is a significant decrease (20%) in the bacterial survival load in the liver and spleen (**Figure 1B**) tissues of mice co-treated with celecoxib and imipenem when compared to imipenem treated mice. Celecoxib treatment alone did not show any effect on the growth of the bacteria.

SIRT1 [sirtuin (silent mating type information regulation 2 homolog) 1 (*S. cerevisiae*)] an NAD-dependent protein deacetylase which regulates expression of various target genes. We have shown previously that celecoxib in combination with ampicillin limits intracellular bacterial infection in macrophages by activating host SIRT1 (Annamanedi and Kalle, 2014). We therefore, analyzed the SIRT1 expression levels in liver and spleen tissues of CLP mice. There was a considerable reduction in the levels of SIRT1 mRNA and protein in CLP group of mice. However, in mice treated with imipenem, celecoxib and combination of both increased the mRNA and protein levels of SIRT1 in the liver and spleen (**Figure 1C**) tissues. There was also a significant increase in the antioxidant enzyme activity of catalase and peroxidase in celecoxib treated and mice receiving both celecoxib and imipenem indicating a protective role of celecoxib-induced SIRT1 in liver and spleen tissues of mice (**Figure 1D**).

#### Regulation of COX-2 by Celecoxib-Activated SIRT1

**FIGURE 2:**
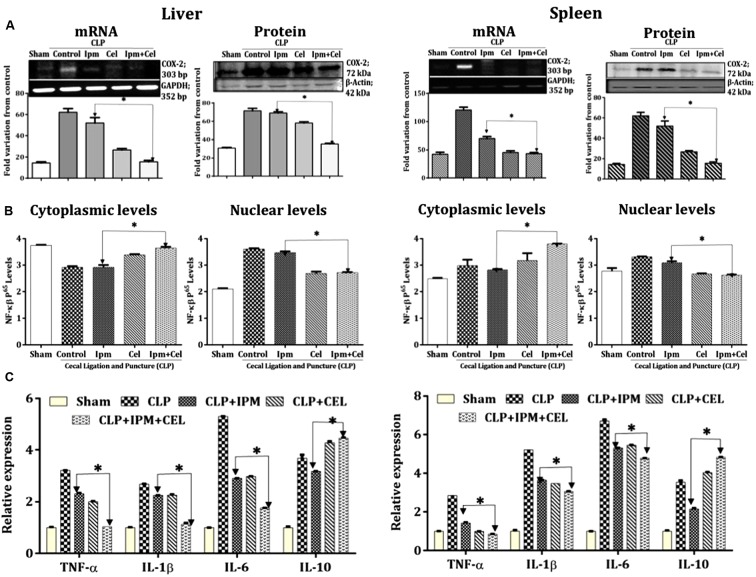
**Regulation of inflammatory response by celecoxib activated SIRT1 *via* COX-2/NF-κB and inflammatory cytokine expression.** **(A)** RT-PCR and Western blot analysis of COX-2 in Sham mice (Lane 1); CLP mice (Lane 2); Imipenem; IPM 5 mg/kg (Lane 3), celecoxib; CEL 5 mg/kg (Lane 4), and combination of celecoxib and imipenem (Lane 5) in the liver tissue (right), and spleen tissue (left), respectively. GAPDH and β-actin served as controls for RT-PCR and Western blot, respectively. The relative band intensities were quantified by ImageJ software. **(B)** NF-κB p65 levels in cytoplasmic and nuclear isolates of the liver tissue and the spleen tissue lysates were determined using ELISA based kit. **(C)** Quantitative real-time PCR showing the mRNA expression levels of TNF-α, IL-1β, IL-6, and IL-10 in liver tissue and spleen tissues. Bars indicate the mean ± SD (*n* = 3); CLP: cecal ligation and puncture. ^∗^*p* < 0.05.

We next analyzed COX-2 protein and RNA levels in liver and spleen tissues. The bacterial infection caused a substantial increase in the COX-2 protein levels and imipenem showed no effect in on COX-2 levels. However, there was a significant decrease in COX-2 levels in celecoxib and combination treatment. The levels of COX-2 are in inverse correlation with the levels of SIRT1 indicating that activated SIRT1 inhibited COX-2 gene transcription and thus protein expression in liver and spleen (**Figure 2A**).

The results from the NF-κB activity assay suggested that SIRT1 activation led to decreased nuclear translocation of NF-κB (p65) in the liver and spleen (**Figure 2B**) tissues of mice treated with imipenem or celecoxib alone and in combination. This further resulted in the decreased pro-inflammatory cytokine gene transcription and increased anti-inflammatory cytokine, IL-10, transcription (**Figure 2C**).

#### Celecoxib Enhanced Antibiotic Efficacy of Ampicillin on Clinical Isolates of ESKAPE Pathogens

**FIGURE 3:**
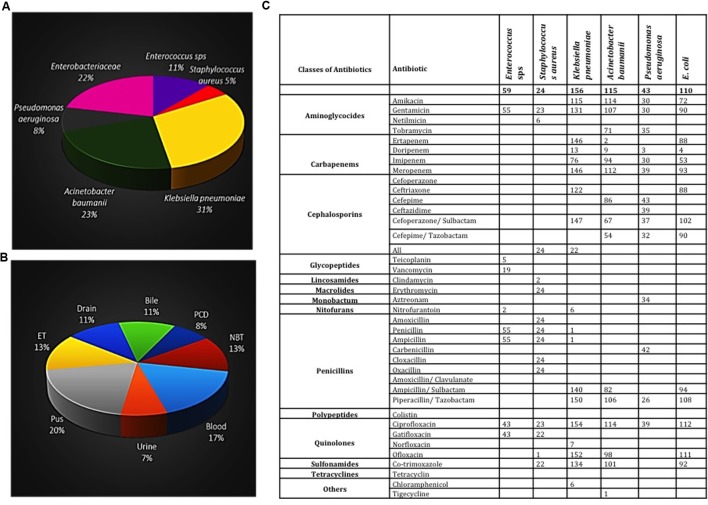
**Details of 507 clinical isolates of ESKAPE pathogens.** **(A)** Pie diagram showing the percentage of each type of bacterial isolates collected. **(B)** The sample source from patients. **(C)** Antibiogram profile of all samples carried out using API automated machine.

Next, we have studied the efficacy of ampicillin and celecoxib combinatorial treatment on clinical isolates of ESKAPE pathogens. A total of 507 samples of ESKAPE pathogens (**Figure 3A**), isolated from various specimens such as pus, blood, urine, drain fluids, etc. of patients (**Figure 3B**), were collected from the hospital. The antibiogram analysis clearly indicated that more than 85% of collected pathogens were resistant to either ampicillin or ampicillin/sulbactam (**Figure 3C**). We have evaluated the ampicillin and celecoxib combinatorial efficacy on ESKAPE pathogens and the results are presented below by organism (**Supplementary Data Sheet 2**).

##### *Enterococcus* sps.

**FIGURE 4:**
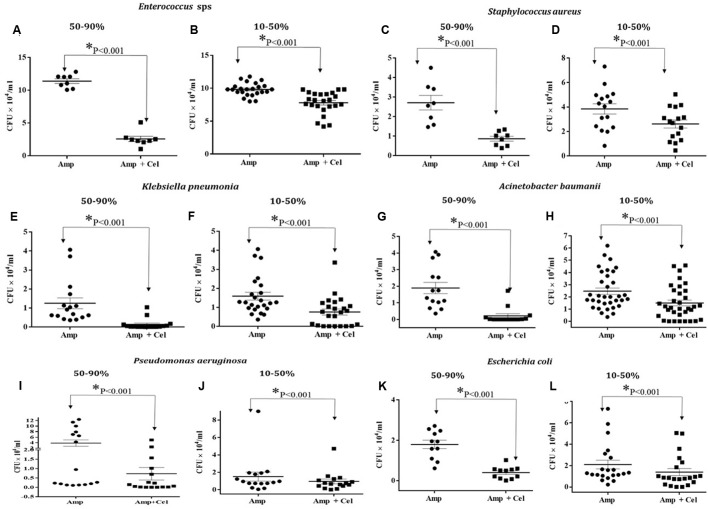
**Effect of Celecoxib and ampicillin co-treatment on the growth of ESKAPE pathogens.** Graphs showing **(A,C,E,G,I,K)** 50–90% reduction **(B,D,F,H,J,L)** 10–50% reduction in the growth (CFU/ml) of clinical isolates of ESKAPE pathogens, respectively, on ampicillin (2 μg/ml) and celecoxib (3.8 μg/ml) containing agar plates when compared to ampicillin (2 μg/ml) alone containing agar plate.

Enterococci cause bloodstream infections, surgical site infections, and urinary tract infections (Rudy et al., 2004). Both *E. faecalis* and *E. faecium* species have emerged as important nosocomial pathogens over the last 30 years and are major hub for the dissemination of antibiotic resistance genes (Guzman Prieto et al., 2016). Fifty-nine strains were received, 39 are *E. faecium*, 16 are *E. gallinarum*, 3 are *E. faecalis*, and 1 is *E. durans.* The 14 *E. gallinarum* isolates and 5 *E. faecium* are resistant to vancomycin. For the present study, randomly 32 samples were tested for ampicillin and celecoxib combinatorial treatment efficacy. The CFU results indicated that 11 out of 32 strains showed 50–90% reduction in CFU/m (**Figure 4A**) and 21 strains showed 10–50% reduction in CFU/mL (**Figure 4B**) on ampicillin (2 μg/ml) and celecoxib (3.8 μg/ml) containing agar plates when compared to ampicillin (2 μg/ml) alone containing agar plate.

##### Staphylococcus aureus

Methicillin-resistant *Staphylococcus aureus* causes skin and wound infections, pneumonia, and blood stream infections that can lead to sepsis and death (Newland and Kearns, 2008). Twenty-four isolates were received from the hospital. All 24 strains were sensitive to vancomycin. Twenty-four samples were tested for ampicillin and celecoxib combinatorial treatment efficacy, 12 strains showed 50–90% reduction in CFU/mL (**Figure 4C**) and 12 strains showed 10–50% reduction in CFU/mL (**Figure 4D**) on ampicillin (2 μg/ml) and celecoxib (3.8 μg/ml) containing agar plates when compared to ampicillin (2 μg/ml) alone containing agar plate.

##### Klebsiella pneumonia

*Klebsiella pneumoniae*, is a Gram-negative facultative anaerobe. causes pneumonia, urinary tract infections, septicaemia, and wound infections (Huang et al., 2015). One hundred and fifty-six isolates were collected among which 22 strains were resistant to all cephalosporins and 12 strains were chloramphenicol resistant. Out of 32 samples tested for ampicillin and celecoxib combinatorial treatment efficacy, 13 strains showed 50–90% reduction in CFU/mL (**Figure 4E**) and 19 strains showed 10–50% reduction in CFU/mL (**Figure 4F**) on ampicillin (2 μg/ml) and celecoxib (3.8 μg/ml) containing agar plates when compared to ampicillin (2 μg/ml) alone containing agar plate.

##### Acinetobacter baumannii

*Acinetobacter* is a type of Gram-negative bacteria resistant for nearly all antibiotics including carbapenems, often considered antibiotics of last resort (Evans et al., 2013). One hundred and fifteen isolates were collected and all the strains are sensitive to tigecycline except one. Thirty-two samples were tested for ampicillin and celecoxib combinatorial treatment efficacy of which 12 strains showed 50–90% reduction in CFU/mL (**Figure 4G**) and 20 strains showed 10–50% reduction in CFU/mL (**Figure 4H**) on ampicillin (2 μg/ml) and celecoxib (3.8 μg/ml) containing agar plates when compared to ampicillin (2 μg/ml) alone containing agar plate.

##### Pseudomonas aeruginosa

*Pseudomonas aeruginosa*, is a Gram-negative opportunistic pathogenic bacterium associated to respiratory, urinary tract, gastrointestinal infections, and bacteraemia in immune compromised patients. RND efflux systems such as the MexXY of *Pseudomonas* is one of the important antibiotic resistance mechanisms (Morita et al., 2014). Forty-three isolates were collected and 32 samples were tested for ampicillin and celecoxib combinatorial treatment efficacy. Eighteen strains showed 50–90% reduction in CFU/mL (**Figure 4I**) and 14 strains showed 10–50% reduction in CFU/mL (**Figure 4J**) on ampicillin (2 μg/ml) and celecoxib (3.8 μg/ml) containing agar plates when compared to ampicillin (2 μg/ml) alone containing agar plate.

##### Enterobacteriaceae

Carbapenem resistant Enterobacteriaceae are causing untreatable blood stream infections (Gupta et al., 2011). Under this group 110 *Escherichia coli* were collected. Of 110 samples, 18 samples were resistant for all cephalosporins and 1 sample was colistin resistant. Thirty-two samples were tested for ampicillin and celecoxib combinatorial treatment efficacy and 18 strains showed 50–90% reduction in CFU/mL (**Figure 4K**) and 14 strains showed 10–50% reduction in CFU/mL (**Figure 4L**) on ampicillin (2 μg/ml) and celecoxib (3.8 μg/ml) containing agar plates when compared to ampicillin (2 μg/ml) alone containing agar plate.

## Discussion

A selection pressure to antibiotics has been created in many of the bacterial strains due to misuse, abuse, and overuse of antibiotics leading to the emergence of new strains with new genotype and proteins (Kolar et al., 2001). Discovery and approval of new antibacterial agents to combat drug resistant infections is a resource consuming effort without a guaranteed elimination of drug resistance (Bollenbach, 2015). Drug repurposing was identified as one of the easy ways to find new antibiotics for emerging resistant bacteria (Brown, 2015). We have reported previously that celecoxib, a non-antibiotic agent in combination with an antibiotic (ampicillin) managed infection and inflammation caused due to *S. aureus* in macrophage model *via* activation of SIRT1 (Annamanedi and Kalle, 2014), a master regulator of inflammation (Salminen et al., 2008). Similarly, an earlier study reported that inflammatory signal transduction inhibitors when combined with antibiotics helped in limiting polymicrobial sepsis and inflammatory responses simultaneously (O’Sullivan et al., 2009).

In the present study, we aimed at evaluating the potency of this combination effect *in vivo* in mouse CLP model of sepsis. The cecum contains a high concentration of microbes which are a combination of Gram-negative and Gram-positive flora (Cuenca et al., 2010). Polymicrobial sepsis induced by CLP is the most frequently used model because it closely resembles the progression and characteristics of human sepsis (Starr et al., 2014). Imipenem was choice of antibiotic in combinatorial treatment with celecoxib, as it is clinically effective in treating various types of Gram-negative and/or Gram-positive bacterial infections and is often recommended for the treatment of polymicrobial intra-abdominal infections (Lipman and Neu, 1988). We have used 5 mg/kg body weight of imipenem, which is less than the dosage generally used in similar experiments (Craciun et al., 2010; Muenzer et al., 2010). It has been well established that CLP induces polymicrobial infection including *Escherichia coli, Streptococcus bovis, Proteus mirabilis, Enterococcus, Bacteroides fragilis*, *Streptococcus viridians* and *Clostridium sporogenes*, etc. (Poli-de-Figueiredo et al., 2008). Our Gram staining results are in agreement with these studies. Uncontrolled bacterial growth is a main cause of death in CLP models. In this study, we showed that celecoxib controlled bacterial growth and inflammatory response efficiently in a murine CLP model of polymicrobial sepsis in combination with imipenem when compared to imipenem alone. These results were in agreement with our *in vitro* study showing that celecoxib induced SIRT1 expression which controlled COX-2 and NF-κB expression resulting in decreased pro-inflammatory cytokine expression (Annamanedi and Kalle, 2014).

Silent information regulator 2 homolog, SIRT1, is known to play a key role in inflammation (Zhang Z. et al., 2010). Studies have shown that pharmacological activation of SIRT1 by resveratrol which helps in resolution of inflammation. SIRT1 has been shown to regulate NF-κB, a key transcriptional regulator of inflammation via inhibition of JNK and ERK1/2 pathway (Lee et al., 2009). SIRT1 is also known to regulate COX-2 gene transcription directly (Zhang R. et al., 2010). COX-2 is known to cause inflammation and pain (Agarwal et al., 2009) and is a direct target of SIRT1. The study results clearly indicate that imipenem cannot effectively regulate the inflammatory response but when used in combination with non-steroidal anti-inflammatory drug (NSAID) such as celecoxib can be effective in limiting bacterial infection even at low doses. NF-κB family of transcription factors play a central part in the host response to infection by microbial pathogens, by orchestrating the innate and acquired host immune responses (Rahman and McFadden, 2011). The activation of NF-κB is increased following infection (Liu X. et al., 2014) and SIRT1 is known to deacetylate NF-κB thereby inhibiting its activity (Shakibaei et al., 2011). Our results are in agreement with earlier studies (Camara-Lemarroy et al., 2015). The results are also similar to earlier studies using imipenem and TAK-242, a toll-like receptor4 inhibitor (Sha et al., 2011).

ESKAPE pathogens (*Enterococcus faecium, Staphylococcus aureus, Klebsiella pneumoniae, Acinetobacter baumannii, Pseudomonas aeruginosa, and Enterobacteriaceae*) are in the priority list for the development of new antibiotics to overcome resistance (Pendleton et al., 2013). Due to evolving antibiotic resistance by these pathogens conventional antibiotics are getting ineffective gradually, hence there is a need for alternate antibacterial approaches (Khan and Khan, 2016). Antibiotics belonging to cephalosporins, carbapenems, β-lactamases inhibitors, aminoglycosides, glycopeptides, tetracyclines, etc. have been shown to be still effective in the drug-resistant ESKAPE pathogens (Bassetti et al., 2013). However, the economic burden on patient as well as country increases with such treatment options (Laxminarayan et al., 2015). Ampicillin, a first generation broad spectrum antibiotic belonging to penicillin-class of antibiotics is now known to be resisted by almost all pathogens. However, it is one of the cheapest and widely available antibiotics till date. Therefore, using ampicillin, at lower MIC, in combination with celecoxib we have shown reversal of antibiotic resistance in laboratory strains of MRSA and *Mycobacterium smegmatis* (Chen et al., 2008). In the present study, we evaluated the efficacy of celecoxib in enhancing ampicillin’s effect even at low concentration of 2 μg/ml on ESKAPE pathogens isolated from various sources of samples of patients. The results are in agreement with our previous results and indicate that a combination therapy using celecoxib and antibiotic might be helpful in reducing the dosage of antibiotic. Furthermore, due to the lack of target for celecoxib in bacteria, development of resistance to celecoxib might be a rare scenario.

## Conclusion

**FIGURE 5:**
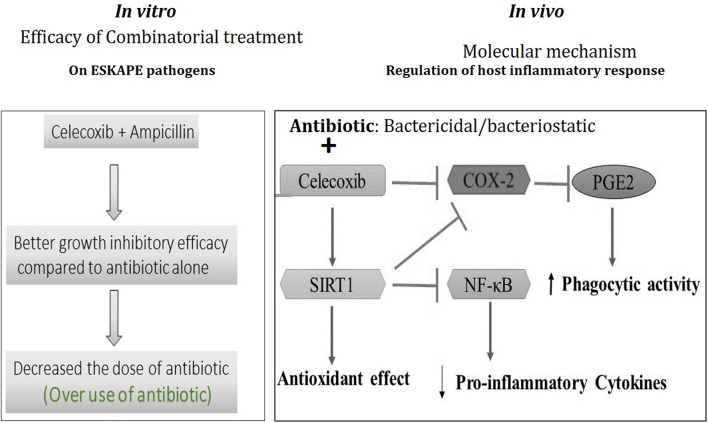
**Schematic representation of the proposed mechanism of action of celecoxib and antibiotic combinatorial treatment.** During bacterial infection, uncontrolled inflammatory response produced by COX-2-PGE2-NF-κB-pro-inflammatory cytokine pathways lead to tissue damage and organ failure. Celecoxib enhances phagocytic efficacy directly by inhibiting COX-2 activity to produce PGE_2_ and regulates host inflammatory response by activating SIRT1. Celecoxib-activated SIRT1 increases antioxidant enzymes and inhibits NF-κB-activated inflammatory cytokines gene expression. Regulated inflammatory response by celecoxib during bacterial infections might create favorable conditions to antibiotics to inhibit bacterial growth in the host. On the other hand, celecoxib also enhances the growth inhibitory efficacy of ampicillin on clinically ampicillin-resistant ESKAPE pathogens at lower concentrations thereby decreasing the dosage of antibiotic.

Our study results imply that celecoxib is effective in controlling inflammatory response by the activation of SIRT1, inhibiting inflammatory protein COX-2, and NF-κB pathways in polymicrobial sepsis murine model and helping antibiotic in clearing the bacterial load. These *in vivo* experimental results are in good association with our *in vitro* data suggesting that celecoxib and antibiotic combinatorial treatment might be a promising treatment strategy to combat drug-resistant bacterial infections. Furthermore, the results from the combinatorial treatment of ampicillin and celecoxib on clinical isolates of ESKAPE pathogens clearly indicated the potential use of this treatment strategy. Based on our earlier and present study, it is proposed that celecoxib acts on host by activating SIRT1 and inhibits proinflammatory markers such as COX-2, NF-κB and cytokines and antibiotic acts on the bacteria and inhibits bacterial growth (**Figure 5**). The results from this study suggest possible implications of use of ampicillin and celecoxib co-treatment in overcoming antimicrobial drug resistance pathogens.

## Ethics Statement

This study was carried out in accordance with the recommendations of Committee for the Purpose of Control and Supervision on Experiments on Animals (CPCSEA) Institutional Animal Ethics Committee (UH/IAEC/AMK/2011-II/5). The protocol was approved by the Institutional Animal Ethics Committee.

## Author Contributions

MA performed immunoblot analysis, cytokine analysis, clinical isolates cfu/ml analysis, and data analysis. GV performed animal experiments. AMK conceptualized, designed, analyzed the data, and drafted the manuscript. AMK collected clinical samples and performed antibiogram. All authors reviewed the manuscript.

## Conflict of Interest Statement

The authors declare that the research was conducted in the absence of any commercial or financial relationships that could be construed as a potential conflict of interest.

## Supplementary Material

**DATA SHEET 1 | Experimental values of *in vivo* results.**

**DATA SHEET 2 | Values of CFU/ml of ESKAPE pathogens formed on agar plates.**
